# Photonic Crystal Surface Mode Real-Time Imaging of RAD51 DNA Repair Protein Interaction with the ssDNA Substrate

**DOI:** 10.3390/bios14010043

**Published:** 2024-01-14

**Authors:** Galina Nifontova, Cathy Charlier, Nizar Ayadi, Fabrice Fleury, Alexander Karaulov, Alyona Sukhanova, Igor Nabiev

**Affiliations:** 1 Laboratoire de Recherche en Nanosciences, LRN-EA4682, Structure Fédérative de Recherche Cap Santé, UFR de Pharmacie, Université de Reims Champagne-Ardenne, 51100 Reims, France; galina.nifontova@univ-reims.fr; 2 Nantes Université, CNRS, US2B, UMR 6286, IMPACT Platform and SFR Bonamy, 44000 Nantes, France; cathy.charlier@univ-nantes.fr; 3 Nantes Université, CNRS, US2B, UMR 6286, DNA Repair Group, 44000 Nantes, France; nizar.ayadi@univ-nantes.fr (N.A.); fabrice.fleury@univ-nantes.fr (F.F.); 4 Department of Clinical Immunology and Allergology, Institute of Molecular Medicine, Sechenov First Moscow State Medical University (Sechenov University), 119146 Moscow, Russia; drkaraulov@mail.ru; 5 Life Improvement by Future Technologies (LIFT) Center, 143025 Moscow, Russia; 6 Laboratory of Nano-Bioengineering, National Research Nuclear University MEPhI (Moscow Engineering Physics Institute), 115522 Moscow, Russia

**Keywords:** one-dimensional photonic crystal, surface mode imaging, microfluidic array, label-free detection, DNA repair protein, RAD51, association–dissociation kinetics

## Abstract

Photonic crystals (PCs) are promising tools for label-free sensing in drug discovery screening, diagnostics, and analysis of ligand–receptor interactions. Imaging of PC surface modes has emerged as a novel approach to the detection of multiple binding events at the sensor surface. PC surface modification and decoration with recognition units yield an interface providing the highly sensitive detection of cancer biomarkers, antibodies, and oligonucleotides. The RAD51 protein plays a central role in DNA repair via the homologous recombination pathway. This recombinase is essential for the genome stability and its overexpression is often correlated with aggressive cancer. RAD51 is therefore a potential target in the therapeutic strategy for cancer. Here, we report the designing of a PC-based array sensor for real-time monitoring of oligonucleotide–RAD51 recruitment by means of surface mode imaging and validation of the concept of this approach. Our data demonstrate that the designed biosensor ensures the highly sensitive multiplexed analysis of association–dissociation events and detection of the biomarker of DNA damage using a microfluidic PC array. The obtained results highlight the potential of the developed technique for testing the functionality of candidate drugs, discovering new molecular targets and drug entities. This paves the way to further adaption and bioanalytical use of the biosensor for high-content screening to identify new DNA repair inhibitor drugs targeting the RAD51 nucleoprotein filament or to discover new molecular targets.

## 1. Introduction

Label-free optical sensors employing fiber optics [1], interferometry [2], surface-enhanced Raman spectroscopy [3], and surface plasmon resonance (SPR) [4] have recently emerged as promising tools for diagnostics, screening, detection of specific proteins, and the analysis of ligand–receptor interactions [5,6,7,8,9]. Photonic crystals (PCs) represent competitive analogues of SPR-based sensors and have been demonstrated to provide ultrasensitive detection of biomolecules of various sizes, such as cancer biomarkers, proteins and peptides, antibodies, and oligonucleotides [10,11,12,13,14,15].

PC-based sensors represent periodic structures in which the spatial distribution of the photonic band gaps and refractive indices of the materials used for their engineering can vary from one to two or three spatial dimensions [16,17]. One-dimensional PCs consist of planar multilayered nanofilm stacks of dielectric materials (silicon dioxide and titanium dioxide) whose properties ensure long-range propagation of optical waves along their surfaces. This enables a higher sensitivity of PC-based sensors compared to those using surface plasmon polaritons [18]. PCs are versatile nanophotonic structures that can be adapted for operation in any wavelength range by tuning their layer thicknesses [19,20,21]. PC surface mode imaging is a novel approach characterized by an enhanced dynamic range and thickness resolution compared to SPR imaging [22]. The imaging of the PC surface mode facilitates the monitoring of multiple binding events that occur at the sensor surface in real time, ensuring the possibility of multiparametric detection [10,23,24]. The PC surface can be functionalized with recognition units, including proteins, with the use of silane chemistry and homo- or heterobifunctional cross-linkers [10,25,26]. Embedding PCs in microfluidic cells and imaging their surface by means of a color camera facilitates real-time analysis in the flow-through mode [10,27].

Proliferative diseases, carcinogenesis, and tumor progression are characterized by genomic instability due to intense DNA damage/DNA repair processes where RAD51 is a key recombinase responsible for homologous recombination. This DNA repair protein is frequently overexpressed by radio- and chemoresistant cancer cells [28,29,30,31] and represents an important prospective biomolecular target for novel anticancer chemotherapeutics inhibiting its activity [29,30,31]. The RAD51 recruitment to single-stranded DNA (ssDNA) allows the formation of the RAD51 nucleoprotein filament which is essential in the first step of homologous recombination pathway [32,33]. Studying the kinetics of the formation of the RAD51–DNA complex and screening the activity of newly developed inhibitors are routinely performed using label-free optical techniques, such as bio-layer interferometry [34,35,36] and classical channel-format SPR [33,37,38], which make it possible to monitor the association–dissociation of the recombinase with its oligonucleotide substrates. However, these approaches have a low throughput and do not provide simultaneous detection of multiple events. Thus, novel sensing approaches, such as PC surface mode imaging, may be relevant for detecting DNA damage/repair biomarkers and screening candidate RAD51 inhibitor anticancer drugs, which are in high demand in modern drug discovery and diagnosis.

This work continues the series of our studies on the development and validation of bioanalytical applications of PC surface mode imaging. Recently, PC surface mode imaging was demonstrated to be effective for multiplexed real-time optical label-free detection of antibody analytes by means of a PC-based protein microfluidic array containing 96 spots of antibodies, proteins A and G, and serum and DNA repair proteins engineered using an automated spotting procedure [10]. Here, we present the first demonstration of this lateral-flow microfluidic approach for highly sensitive optical label-free detection and simultaneous monitoring of multiple events of association–dissociation of the model DNA repair protein (RAD51) to/from its oligonucleotide substrate. This technique provides real-time data on the interaction of this recombinase with oligonucleotide without additional labelling or signal amplification. We also describe the results of optimizing the chemical modification of the sensor surface with protein recognition units to ensure its reliable response and the proof-of-concept study on the simultaneous detection of 32 target association–dissociation profiles in a model recombinase–oligonucleotide array. The new data pave the way to further optimization of the approach and engineering of a sensor to be used in high-content screening.

## 2. Materials and Methods

### 2.1. Materials

Neutravidin (NeutrAvidin), 1,4-dithiothreitol (Cleland’s reagent, DTT) were obtained from the Thermo Fisher Scientific, Illkirch, France. Goat anti-mouse IgG (whole molecule), adenosine 5′-triphosphate magnesium salt (ATP), and bovine serum albumin (BSA) were purchased from the Merck Group, Saint-Quentin-Fallavier, France. The model oligonucleotide substrate consisting of 80 thymidine bases and biotinylated at the 5′ end with a molecular weight of ~24.6 kDa (polydT(80)–biotin) was supplied by Eurofins Genomics, Ebersberg, Germany.

Human RAD51 was prepared and purified as described earlier [34]. Briefly, after cloning cDNA of His-RAD51 into the pET15a vector (Novagen, Merck Group, Saint-Quentin-Fallavier, France), RAD51 was expressed in *Escherichia coli* BL21-DE3 strain at 37 °C. RAD51-His proteins were purified using nickel-charged NiNTA affinity resin (Invitrogen, Thermo Fisher Scientific, Waltham, MA, USA). Protein quantification was performed using the BCA assay. The purity of the proteins was analyzed by SDS-PAGE. The RAD51 protein was kept in Tris-based storage buffer (20 mM Tris-HCl (pH 8), 200 mM KOAc (pH 8), 10% glycerol, 1 mM EDTA, 0.5 mM DTT, and sterile water q.s.) at −80 °C until use.

For PC surface modification and activation, glutaraldehyde and 3-(aminopropyl)triethoxysilane (APTES) purchased from Merck Group, Saint-Quentin-Fallavier, France were used. Absolute ethanol, absolute acetone, and the other reagents for preparation of all the buffer solutions used in the study were of analytical grade. The buffer solutions were prepared using ultrapure water (Milli-Q; purity grade, 18.2 mΩ·cm) obtained by means of a Direct-Q water purification system (Merck Group, Saint-Quentin-Fallavier, France) and additionally filtered through sterile filters with a PVDF membrane, with a pore size of 0.22 μm.

### 2.2. Photonic Crystal Aminosilanization and Surface Activation

**Figure 1:**
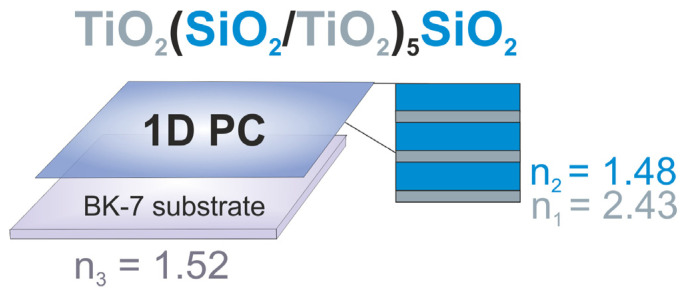
Schematic representation of the photonic crystal used for engineering a microfluidic array. Abbreviations: 1D PC, one-dimensional photonic crystal; n_1_, refractive index of the titanium dioxide layer; n_2_, refractive index of the silicon dioxide layer; n_3_, refractive index of the BK-7 glass substrate.

The one-dimensional PCs were engineered as shown in Figure 1 by means of electron-beam evaporation and plasma ion-assisted deposition of alternating titanium dioxide (TiO_2_, n_λ 500 nm_ = 2.43; layer thickness, 64 nm) and silicon dioxide (SiO_2_, n_λ 500 nm_ = 1.48; intermediate layer thickness, 214.3 nm; final layer thickness, 298.8 nm) layers until a TiO_2_(SiO_2_/TiO_2_)_5_SiO_2_ multilayered stack was formed at the surface of the BK-7 glass substrate (n_λ 500 nm_ = 1.52), as described earlier [10,22,23,24].

Before the modification, the sensor surface was cleaned by washing with absolute ethanol and sonicated using an ultrasonic bath for 10 min. The cleaning cycle was repeated using absolute acetone. Afterwards, each side of the sensor was treated by means of an UV-cleaning device for 45 min. The precleaned PC was incubated in a 1% acetone solution of APTES overnight at room temperature. After the incubation, the PC was washed thrice with absolute acetone for 10 min to remove excess APTES. Then, the PC was baked at 120 °C for 90 min, and its surface was activated directly before the spotting procedure via a 30 min incubation in a 2.5% glutaraldehyde solution in 0.02 M phosphate buffer (pH 7.4). After incubation, excess cross-linker was removed by washing with 0.02 M phosphate buffer (pH 7.4), and excess buffer was removed from the sensor surface by air purging.

### 2.3. Deposition of Protein Recognition Units

Neutravidin and anti-mouse IgG antibody were used as the recognition units of the sensor and were applied by means of a sciFLEXARRAYER S3 with a Piezo Electric Dispenser (Scienion, Berlin, Germany). Then, 0.45 nL drops containing 0.25, 0.5, 1, 1.5, and 2 mg/mL neutravidin solutions in 0.02 M phosphate buffer (pH 7.4) and 0.099, 0.198, 0.4, 0.6, and 0.8 mg/mL anti-mouse IgG solutions in 0.02 M phosphate buffer (pH 7.4) were applied onto the preliminary glutaraldehyde-activated APTES-coated PC surface in 1, 2, 5, and 10 drops per spot in duplicates. The spots were formed within 150 mm^2^ areas of the surface of the pre-functionalized PC at 25 °C and a humidity of 60%. The procedure was controlled by a live stream camera. The prepared PC with the deposited protein pattern was dried and stored at 4 °C for no longer than 1.5 months until use.

Neutravidin and IgG spots were deposited in the same manner using two parts of the array to allow analysis of the specificity of the interactions observed and to eliminate the possibility of spot smearing. The neutravidin spots served for immobilizing biotinylated ssDNA to further monitor RAD51 association–dissociation. The IgG units deposited served as reference spots to determine the specificity of binding of the model recombinase and its substrate to the sensor surface since IgG was not supposed to interact specifically with polydT(80)-biotin. The referencing strategy employed was based on the analysis of the signals recorded within the reference (IgG) spots and its subtraction from the sensorgrams recorded over the neutravidin spots.

### 2.4. Photonic Crystal Surface Mode Imaging

In order to ensure imaging of the prepared PC-based array and analysis of biomolecule association, the PC was mounted onto a configured Kretschmann-like prism of an optimized EVA 3.0 device [10,22]. A +45° polarized parallel light beam (λ = 500 nm) illuminated the PC, which maintained p-polarized surface modes at the specified wavelength, through a prism (Figure S1, Supplementary Materials). The light reflected by the prism came through the second polarizer (−45°) and was recorded by the color camera. The optical signal resulting from the changes in the adsorption layer thickness and the position of the PC reflectance peak was then converted and normalized as the ratio of the blue and green pixel values at each point of the camera, which was recalculated as the shift Δλ (equations used for data processing are presented in the Experimental Part, Supplementary Materials) [22]. After the PC was mounted, a single channel microfluidic chamber was assembled and connected to an Ismatec Reglo ICC computer-operated peristaltic pump (Washington, DC, USA), which maintained the flow rate of 30 μL/min. Using the device control software, the sensor surface was imaged, and the regions of interest corresponding to the protein spots were selected in the image (Figure S2, Supplementary Materials). In this way, multiple signals from the PC surface were simultaneously recorded and monitored over time.

### 2.5. Oligonucleotide–RAD51 Association

The cell was equilibrated by injecting a 0.02 M phosphate buffer (pH 7.4) and the blocking buffer (10 mg/mL BSA in 0.02 M phosphate buffer, pH 7.4) for 600 s each. Afterwards, the running buffer was injected for 300 s. Then, the biotinylated oligonucleotide was immobilized by running a 50 μM solution of polydT(80)–biotin through the cell for 500 s. After modification of the PC surface with the biotinylated oligonucleotide in the flow mode, the interaction of polydT(80)–biotin and RAD51 was analyzed. Initially, the reaction buffer (phosphate-buffered saline (pH 7.2) containing 2 mM ATP and 2 mM DTT) was introduced into the cell for 600 s. Then, the equilibration buffer was injected for 600 s. This was the reaction buffer containing the same amount of glycerol as the prepared dilutions in order to diminish refractive index changes. Finally, 0.001–5 μM solutions of RAD51 in the reaction buffer were injected for 500–600 s each until the signal stabilization.

### 2.6. Data Analysis and Statistical Treatment

To obtain statistics on sensor bioanalytical characteristics the data were monitored over the duplicated spots of protein recognition units. Primary processing, including data classification, was performed using the MS Office Excel 2021 software. The resultant sensorgrams are presented as the mean of two sensorgrams recorded over the corresponding spots. Subtraction of the reference data recorded over neutravidin/IgG spots containing the same amount of proteins, and normalization of the datasets on the obtained sensorgrams or their fragments by dividing the recorded dataset by the maximum value (da_max_) determined for the selected time frames were performed using the Origin Pro version 2018 software (OriginLab Corporation, Northampton, MA, USA). 

## 3. Results and Discussion

### 3.1. Modification of the Surface of a Photonic Crystal–Based Sensor with Protein Recognition Units

**Figure 2:**
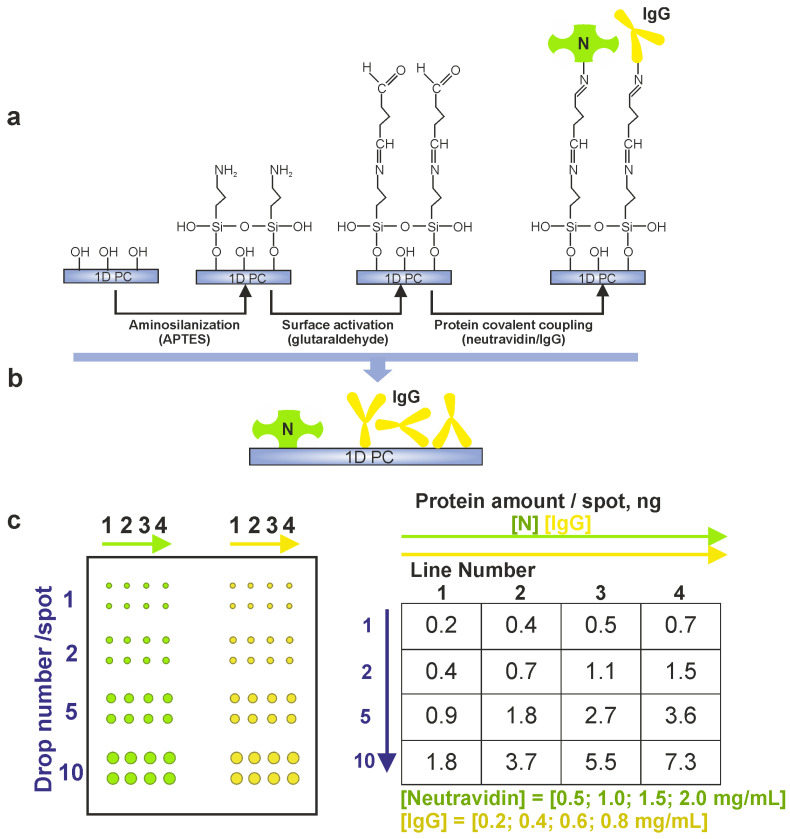
Surface design of the photonic crystal–based sensor for analysis of RAD51–oligonucleotide association. (**a**) Surface modification of the photonic crystal. (**b**) Orientation of the protein recognition units (neutravidin and the control immunoglobulin G) after covalent coupling to the photonic crystal surface. (**c**) The schematics of the protein array and the amounts of proteins deposited per spot. Neutravidin spots are shown with green frames; control protein spots are shown with yellow frames. The spots were applied as duplicated lines. Abbreviations: 1D PC, one-dimensional photonic crystal; APTES, (3-aminopropyl)triethoxysilane; IgG, immunoglobulin G; N, neutravidin.

Proteins of the avidin family are widely used for surface modification of the sensing interface to ensure oriented attachment and steric availability of biotinylated ligands [39]. Neutravidin has been demonstrated to be suitable for immobilization of oligonucleotides [40], ensuring steric availability of at least two biotin-binding sites for further binding of a model biotinylated oligonucleotide to be immobilized on the sensor surface. The use of neutravidin ensures the best cooperativity in the binding of biotin or biotinylated substrates and lower nonspecific binding due to modifications of its positively charged groups and more neutral pI (~6.3) compared to streptavidin [41,42]. To allow further analysis of RAD51–oligonucleotide association kinetics, neutravidin and the control goat anti-mouse IgG were used as protein recognition units of the sensor (Figure 2). In order to ensure the monitoring of multiple binding events, neutravidin and the control protein (IgG) were deposited onto a preactivated surface of the PC as an array of 64 spots [10,43].

Both IgG and neutravidin were covalently coupled to the PC surface preliminary modified with APTES through glutaraldehyde cross-linking. Glutaraldehyde is a homofunctional bivalent crosslinker ensuring conjugation via primary amine groups of APTES deposited at the PC surface and proteins. After spotting protein solutions onto the preactivated surface of the sensor, intermediary Schiff bases were formed, which enabled further covalent coupling of the proteins via primary amine residues (Figure 2a,b) [44,45,46].

The amounts of neutravidin and the control IgG in each spot were the same and gradually increased from spot to spot (from 0.2–0.7 ng to 1.8–7.3 ng in the whole pattern, Figure 2c). The difference in molecular size/weight between neutravidin (~60 kDa) and IgG (~150 kDa) [47,48] and the distribution of the protein solution on the activated PC surface (drop volume per 1 mm^2^ of the spot area) were taken into account to ensure the formation of a uniform protein film after the drop was applied. Protein mass transfer due to spot smearing upon the assembly of the microfluidic cell and injections of running buffers was prevented by keeping a distance of 2.7 mm between the neutravidin and control IgG spots.

### 3.2. Surface Blocking and Immobilization of the Model Oligonucleotide

After preparation of the protein array, the PC was placed onto the prism and fixed in a microfluidic cell through which running and blocking buffers were subsequently injected until signal saturation. As-recorded sensorgrams illustrating the preparation of the PC surface, assembly of the bioanalytical complex, and monitoring of the RAD51–oligonucleotide association are presented in Figure S3, Supplementary Materials.

The BSA blocking step was performed to minimize undesired cross-linking of the target analyte (RAD51) and nonspecific binding of the reagents to be introduced into the microfluidic cell [49,50]. The running buffer was re-injected to remove poorly adsorbed BSA and to prevent excessive mass transfer of the blocking protein and ensure effective binding of the model biotinylated oligonucleotide, polydT(80)–biotin consisting of 80 thymidine bases biotinylated at the 5′ end. PolydT(80)–biotin was chosen as a model substrate due to the known quick RAD51 polymerization on ssDNA substrates, which are more flexible than dsDNA [33]. PolydT(80) is characterized by a high flexibility only slightly affected by steric hindrance due to base residues, which makes it an appropriate substrate for monitoring RAD51 association–dissociation profiles [33,51].

**Figure 3:**
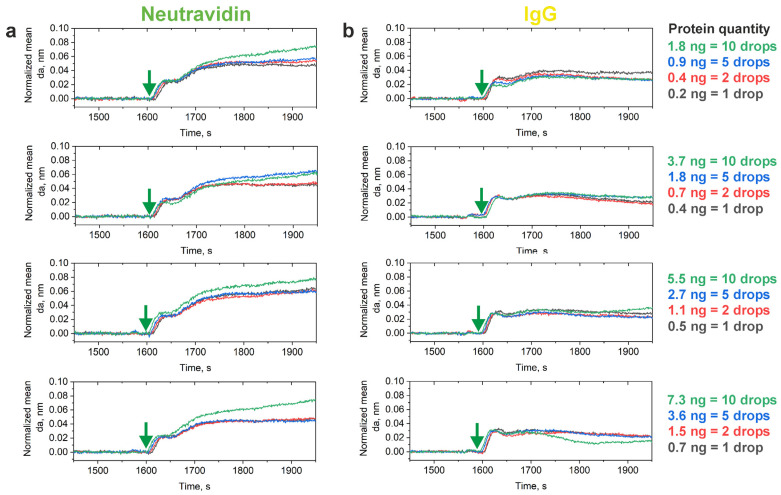
Fragments of normalized sensorgrams recorded during injection of the model biotinylated oligonucleotide (a 50 µM solution of polydT(80)–biotin) in the areas corresponding to the (**a**) neutravidin and (**b**) control immunoglobulin G spots. The start of oligonucleotide solution injection is indicated by green arrows. The legend specifies the quantities of neutravidin and control IgG deposited in the corresponding parts of the protein array. Each sensorgram is an average of two sensorgrams recorded in duplicate. Abbreviation: IgG, immunoglobin G.

The fragments of the sensorgrams of polydT(80)–biotin binding within the deposited protein spots are shown in Figure 3. The normalized curves indicate intense attachment of the polydT(80)–biotin complex to the neutravidin spots. The gradual increase in the adlayer thickness upon running the polydT(80)–biotin solution indicate effective continuous immobilization of the model oligonucleotide at the target spots, which was more pronounced in the areas corresponding to 5 and 10 drops of the protein solution per spot. A shift in the adlayer thickness change (Δda) after the injection of the oligonucleotide solution did not depend significantly on the quantities of neutravidin and IgG in the spots (Table S1, Supplementary Materials). This shift can be attributed to the bulk shift when the 50 µM solution of polydT(80)–biotin was run. However, the slope of the sensorgrams recorded over the neutravidin spots was greater compared with the control protein (IgG) spots, where oligonucleotide traces may have caused rapid saturation of the sensor response due to nonspecific interactions, and the maximum adlayer thickness gain was significantly smaller (Table 1). The maximum gains of adlayer thickness recorded over proteins spots differed significantly (*p* < 0.05, Student’s *t* test), the adsorption and immobilization of the biotinylated oligonucleotide in the neutravidin spots being more intense and specific (Table 1).

### 3.3. Analysis of RAD51–Oligonucleotide Association

Analysis of RAD51 recruitment was performed in the titration mode by gradually increasing the RAD51 concentration in the solutions injected into the microfluidic cell and monitoring the adlayer thickness changes in the neutravidin and control IgG spots. After polydT(80)–biotin immobilization, the reaction buffer, equilibration buffer, and 0.001 to 5 µM solutions of RAD51 in the reaction buffer containing 2 mM dATP were sequentially injected into the microfluidic cell until the sensor response and signal saturation occurred (Figure S3, Supplementary Materials). The equilibration buffers also contained various amounts of glycerol corresponding to that in the RAD51 dilutions. The equilibration buffer was used to prevent bulk shifts during titration of the RAD51 dilutions.

**Figure 4:**
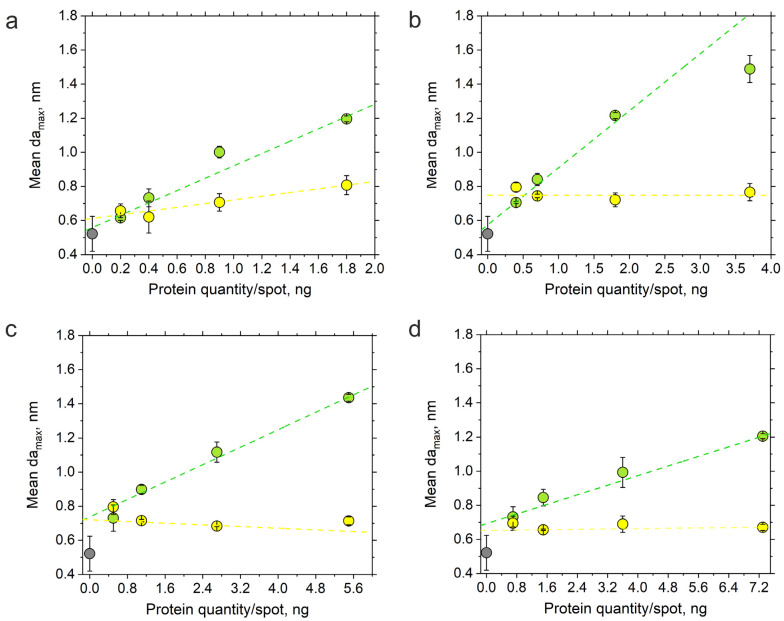
The sensor response as a function of the amount of neutravidin deposited onto the photonic crystal surface after running all the RAD51 dilutions (from 0.001 µM to 5 µM): (**a**) line 1 (0.2, 0.4, 0.9 and 1.8 ng of neutravidin); (**b**) line 2 (0.4, 0.7, 1.8 and 3.7 ng of neutravidin); (**c**) line 3 (0.5, 1.1, 2.7 and 5.5 ng of neutravidin); (**d**) line 4 (0.7, 1.5, 3.6 and 7.3 ng of neutravidin). The mean values of maximum gains of the adlayer thickness recorded over the neutravidin spots are shown with green dots; those recorded over the control immunoglobulin G (IgG) spots are shown with yellow dots; those recorded over the reference areas of the photonic crystal surface that did not contain protein recognition units are shown with gray dots. Each dot shows the mean values of maximum gains in the adlayer thickness of two sensorgrams recorded in duplicate.

**Figure 5:**
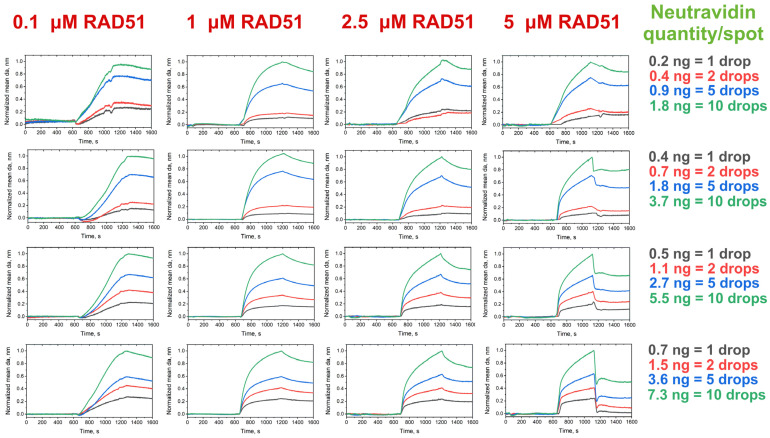
Association–dissociation profiles of the RAD51 to a model oligonucleotide substrate recorded in the neutravidin spots. The values shown are obtained by subtracting the blank sensorgrams recorded over the control immunoglobulin G spots from those recorded over the spots with the same amount of neutravidin. Each sensorgram is an average of two sensorgrams recorded in duplicate and additionally normalized.

The most prominent PC response was observed when 0.1 µM or more of RAD51 was introduced into the microfluidic cell. An increase in the introduced RAD51 concentration resulted in greater changes in the adlayer thickness over the neutravidin part of the protein pattern. On the black-and-white images of the pattern recorded in the beginning of the experiment and after the injection of all RAD51 samples tested, the spots corresponding to the neutravidin part of the protein pattern were clearly revealed, which indicated considerable adlayer thickness gains in the spots (Figure S4, Supplementary Materials). However, the possible presence of oligonucleotide traces or nonspecific adsorption of RAD51 led to a weak response in the corresponding control IgG spots (Figure 4). Thus, the sensorgrams recorded over the control spots were used as blanks to consider the contribution of nonspecific binding to the adlayer thickness gain in neutravidin spots (Figure S5, Supplementary Materials). By subtracting and normalizing the data, we derived the profiles of RAD51–polydT(80)–biotin association–dissociation (Figure 5). RAD51–oligonucleotide association was observed even in the smallest spots (1 drop/spot). Running of the 0.1 µM RAD51 solution ensured the monitoring of the initial RAD51 polymerization; however, introduction of higher RAD51 concentrations (from 1 to 5 µM) resulted in fast polymerization of RAD51 on polydT(80)–biotin, resulting in signal plateauing and rapid dissociation of the complex when the equilibration or running buffer was injected (Figure 5).

### 3.4. Limit of Detection and Linearity of the Sensor Response

The baseline noise was estimated as the standard deviation of the baseline thickness due to the injection of the first equilibration buffer used to diminish the possible bulk shifts before RAD51 titration. It was determined to be 6.3 × 10^−4^ nm. The signal-to-noise ratios (S/N) were calculated from the changes in the adlayer thickness after the injection of at least 0.1 μM RAD51 (the maximum adlayer thickness gain) recorded in the neutravidin spots corresponding to 1 and 10 drops. These values were found to be 70 and 250, respectively. The RAD51 limit of detection (LoD) was taken to be its concentration at which S/N = 3. It was found to be 5 nM (the average value for the entire neutravidin array). Indeed, the analysis of RAD51–polydT(80)–biotin association profiles showed that injection of 0.01 μM RAD51 led to a poorly detectable adlayer thickness shift, and the RAD51 LoD was about 10 nM (Figure S6, Supplementary Materials). However, due to the small adlayer thickness changes, measurements in the LoD range did not provide reliable data on RAD51 recruitment. Thus, the minimum RAD51 concentration should be at least 0.1 μM to ensure distinct adlayer thickness changes and association–dissociation of the RAD51–oligonucleotide complex.

The linearity analysis of the maximum gain of the mean adlayer thickness after the introduction of the 0.1 μM RAD51 solution showed that the observed signal at the introduction of the analyte concentration under study rose with an increase in the neutravidin quantity in lines 1–4. At the same time, adjusted *R*^2^ values close to 1 were observed in line 3 (0.5, 1.1, 2.7 and 5.5 ng of neutravidin per spot) and line 4 (0.7, 1.5, 3.6 and 7.3 ng of neutravidin per spot) (Figure S7, Supplementary Materials). Deposition from 0.5 to 5.5 ng and from 0.7 to 7.3 ng of neutravidin per spot provided the lowest drift and most reliable sensor response (Figure 5). Hence, the application of more concentrated neutravidin spots ensured a more linear sensor response in detecting and analyzing RAD51–oligonucleotide association in the given concentration range.

Thus, the observed profiles are similar to those obtained using the classical SPR technique; the minimum RAD51 quantities detectable by both methods are in about the same range, 0.10–0.15 µM [33]. Quartz crystal microbalance with dissipation monitoring has been also employed for real-time detection of RAD51 within the nanomolar concentration range. However, RAD51 sensing using this technique is time-consuming and characterized by a continuous sensor response [52]. Other similar label-free optical approaches, such as BLItz, are low-cost, easy to use, but less sensitive, with the RAD51–oligonucleotide association reliably detected only at a concentration no lower than 1 µM and can be used only for low-throughput screening [53,54]. The use of PC-based protein arrays ensures highly sensitive detection within the femtogram range, as it was previously demonstrated for antibody (IgG) analytes. However, in the case of RAD51, the detectable range has been found to be at the picogram level, which is three orders of magnitude lower. This could be explained by the difference between the molecular weights of the analytes (RAD51, ~37 kDa; IgG, ~150 kDa) [48,55] or by the complex stoichiometry of cooperative interaction with the recognizing elements of the sensor leading to the changes in the adlayer thickness recorded by the system [33,56]. Therefore, the use of the PC-based protein array and PC surface mode imaging developed here provides a more sensitive, easy, and rapid label-free optical detection of the small-sized RAD51 protein compared with the mentioned approaches and allows the ultrasensitive analysis of its association–dissociation with/from the model oligonucleotide substrate in picogram amounts.

## 4. Conclusions

Thus, we have demonstrated that PCs can be effectively used for simultaneous real-time label-free optical detection and monitoring of the RAD51–oligonucleotide association–dissociation profiles. The application of spots of protein recognition units, neutravidin and the control IgG, onto the PC surface, followed by immobilization of the model biotinylated oligonucleotide (polydT(80)–biotin) and titration with RAD51 dilutions in the flow mode, made it possible to detect at least 32 association–dissociation events with a high sensitivity, without signal amplification in the real-time mode. The sensor response to RAD51 injections into the microfluidic cell was observed in the neutravidin spots. This proved the effective attachment of polydT(80)–biotin and polymerization of RAD51 on its substrate. The optimal neutravidin quantity per spot ensuring reliable recordings of the sensor response and clear sensorgrams varied from 0.5 to 5.5 ng and from 0.7 to 7.3 ng per spot. In this case, the linearity of the signal response was higher (with *R*^2^ close to 1) than in the case of picogram amounts of RAD51. The approach developed here provides a better sensitivity of RAD51 binding analysis compared with the label-free Blitz and classical SPR. Our data pave the way to further optimization of the approach and engineering of a sensor for high-content screening for new DNA repair inhibitors. The obtained results demonstrate a distinct potential of the new technique to be applied to testing the functionality of candidate drugs, discovering new molecular targets and drug entities, detecting RAD51 forms, and diagnosing malignancies.

## Figures and Tables

**Table 1:** Maximum gain of normalized adlayer thickness (da_max_) over the neutravidin/control immunoglobulin G (IgG) spots after running of the model biotinylated oligonucleotide (a 50 µM solution of polydT(80)–biotin).

Protein Quantity/Spot, ng	da_max_ in Neutravidin Spots *, nm	da_max_ in IgG Spots *, nm
0.2 (1 drop)	0.0503	0.0405
0.4 (2 drops)	0.0553	0.0363
0.9 (5 drops)	0.0586	0.0341
1.8 (10 drops)	0.0754	0.0317
0.4 (1 drop)	0.0485	0.0332
0.7 (2 drops)	0.0497	0.0319
1.8 (5 drops)	0.0663	0.0342
3.7 (10 drops)	0.0637	0.0355
0.5 (1 drop)	0.0655	0.0347
1.1 (2 drops)	0.0635	0.0297
2.7 (5 drops)	0.0626	0.0306
5.5 (10 drops)	0.0796	0.0370
0.7 (1 drop)	0.0487	0.0335
1.5 (2 drops)	0.0491	0.0290
3.6 (5 drops)	0.0467	0.0320
7.3 (10 drops)	0.0749	0.0317

* Differences are significant for all specified quantities of neutravidin and control IgG per spot (*p* < 0.05, Student’s *t* test).

## Data Availability

The datasets generated during and/or analyzed during the current study are available from the corresponding author on reasonable request.

## Supplementary Materials

The following supporting information can be downloaded at: https://www.mdpi.com/article/10.3390/bios14010043/s1, Figure S1: The operation scheme of the microfluidic assay for label-free real-time monitoring of oligonucleotide–RAD51 association kinetics based on photonic crystal surface mode imaging; Figure S2: The layout of the selected regions of interest for sensor response monitoring, including 64 areas corresponding to duplicates of the deposited spots; Figure S3: Recorded sensorgrams of RAD51–oligonucleotide association in the selected spots; Figure S4: Protein spot images before and during titration with RAD51; Figure S5: Sensorgrams of RAD51–oligonucleotide association in the selected spots after blank subtraction; Figure S6: Representative sensorgrams demonstrating RAD51–oligonucleotide association in the selected spots after running solutions containing the RAD51 amount close to the limit of detection; Figure S7: Linearity of the sensor response as a function of the amount of neutravidin applied onto the photonic crystal surface; Table S1: Changes in the normalized mean adlayer thickness values (Δda) in neutravidin and immunoglobulin G spots after running the oligonucleotide solution.
