# Supplementary material for: Photonic Crystal Surface Mode Real-Time Imaging of RAD51 DNA Repair Protein Interaction with the ssDNA Substrate
Source: Biosensors (Basel). 2024 Jan 14;14(1):43. doi: 10.3390/bios14010043 (PMC10813746; doi:10.3390/bios14010043)
Supplement: Supplementary file 1 [file biosensors-14-00043-s001.zip › biosensors-2738462-supplementary.pdf]

Supplementary Materials

Photonic Crystal Surface Mode Real-Time Imaging of RAD51 DNA Repair Protein Interaction with the ssDNA Substrate

Galina Nifontova [1], Cathy Charlier [2], Nizar Ayadi [3], Fabrice Fleury [3], Alexander Karaulov [4], Alyona Sukhanova [1,5,*] and Igor Nabiev [1,4,5,6,*]

[1] Laboratoire de Recherche en Nanosciences, LRN-EA4682, Structure Fédérative de Recherche Cap Santé, UFR de Pharmacie, Université de Reims Champagne-Ardenne, 51100 Reims, France; galina.nifontova@univ-reims.fr
[2] Nantes Université, CNRS, US2B, UMR 6286, IMPACT Platform and SFR Bonamy, 44000 Nantes, France; cathy.charlier@univ-nantes.fr
[3] Nantes Université, CNRS, US2B, UMR 6286, DNA Repair group, 44000 Nantes, France; nizar.ayadi@univ-nantes.fr (N.A.); fabrice.fleury@univ-nantes.fr (F.F.)
[4] Department of Clinical Immunology and Allergology, Institute of Molecular Medicine, Sechenov First Moscow State Medical University (Sechenov University), 119146 Moscow, Russia; drkaraulov@mail.ru
[5] Life Improvement by Future Technologies (LIFT) Center, 143025 Moscow, Russia
[6] Laboratory of Nano-Bioengineering, National Research Nuclear University MEPhI (Moscow Engineering Physics Institute), 115522 Moscow, Russia
* Correspondence: alyona.sukhanova@univ-reims.fr (A.S.); igor.nabiev@univ-reims.fr (I.N.)

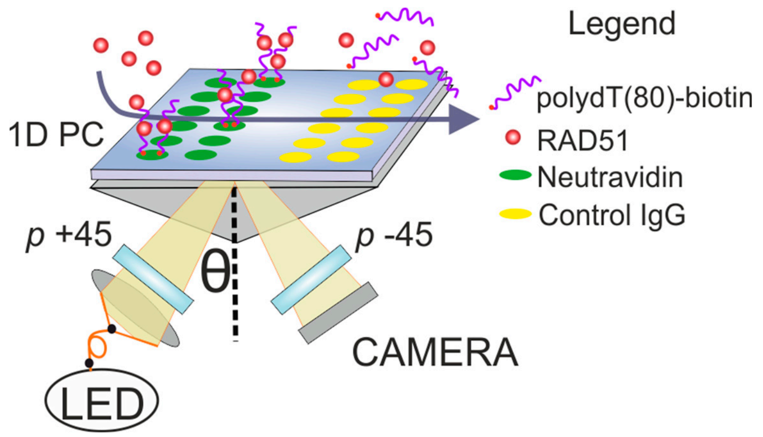

Figure S1. The operation scheme of the microfluidic assay for label-free real-time monitoring of oligonucleotide–RAD51 asscociation based on photonic crystal surface mode imaging. Abbreviations: polydT(80)-biotin, model biotinylated oligonucleotide; RAD51, DNA repair protein; IgG, immunoglobulin G; 1D PC, one-dimensional photonic crystal.

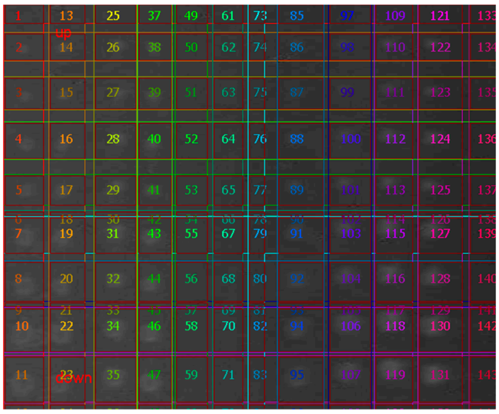

Figure S2. The layout of the selected regions of interest for sensor response monitoring, including 64 areas corresponding to duplicates of the deposited spots.

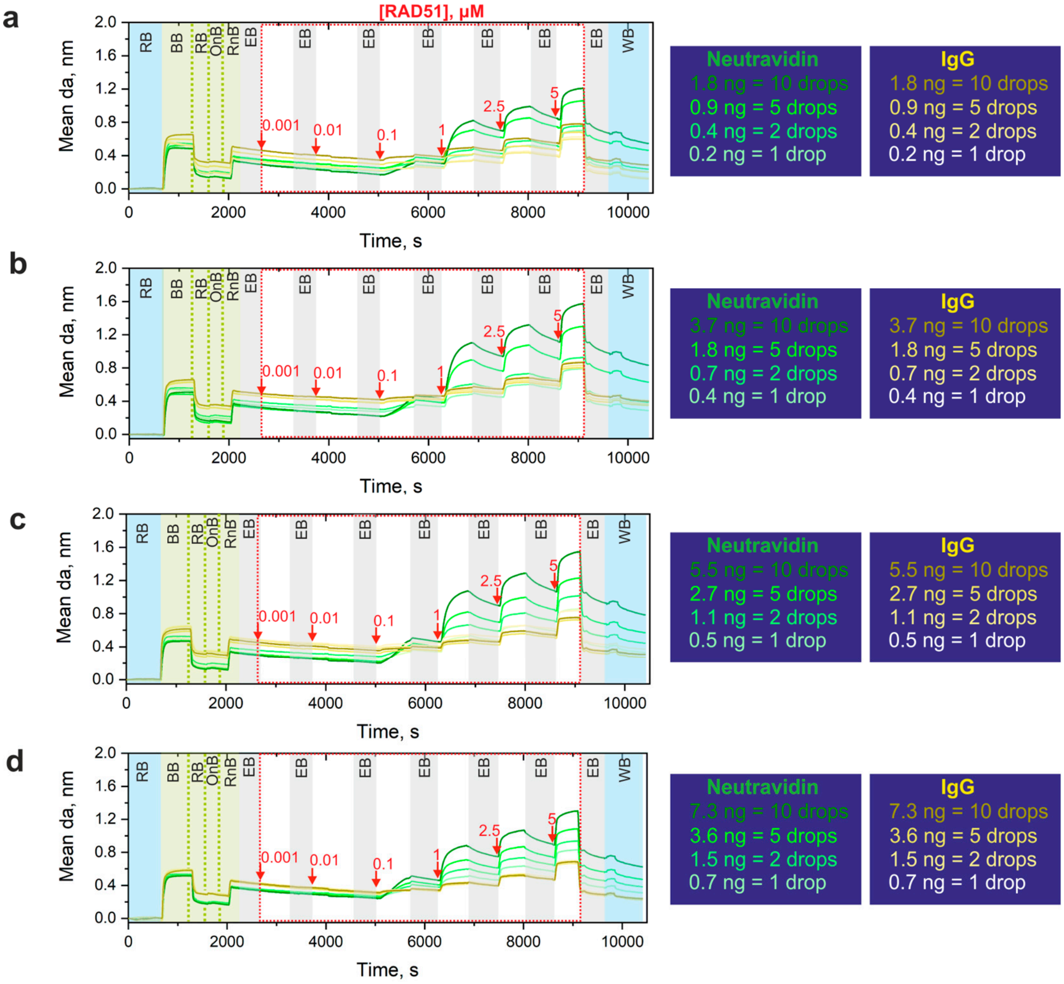

Figure S3. Recorded sensorgrams of RAD51–oligonucleotide association in the selected spots. The start of the subsequent injections of each concentration of RAD51 is indicated with an arrow. Each sensorgram is an average of two sensorgrams recorded in duplicate. **(a)** line 1 (0.2, 0.4, 0.9, and 1.8 ng of protein per spot); **(b)** line 2 (0.4, 0.7, 1.8, and 3.7 ng of protein per spot); **(c)** line 3 (0.5, 1.1, 2.7, and 5.5 ng of protein per spot); **(d)** line 4 (0.7, 1.5, 3.6, and 7.3 ng of protein per spot). Abbreviations: RB, running buffer; BB, blocking buffer; OnB, 50 µM solution of polydT(80)–biotin; RnB, reaction buffer; EB, equilibration buffer; [RAD51], the 0.001 µM, 0.01 µM, 0.1 µM, 1 µM, 2.5 µM, and 5 µM dilutions of the DNA repair protein; WB, washing buffer.

Table S1. Shifts of the normalized adlayer thickness values (Δda) in neutravidin and immunoglobulin G spots after running the oligonucleotide solution

Protein quantity/spot, ng	Δda in neutravidin spots, nm	Δda in immunoglobulin G spots, nm
0.2 (1 drop)	0.0167*	0,0322*
0.4 (2 drops)	0.0198*	0,0294*
0.9 (5 drops)	0.0231*	0,0213*
1.8 (10 drops)	0.0223*	0,0163*
0.4 (1 drop)	0.0217**	0,0274**
0.7 (2 drops)	0.0228**	0,0261**
1.8 (5 drops)	0.0257**	0,0242**
3.7 (10 drops)	0.0192**	0,0281**
0.5 (1 drop)	0.0217***	0,0302***
1.1 (2 drops)	0.0228***	0,0269***
2.7 (5 drops)	0.0237***	0,0263***
5.5 (10 drops)	0.0279***	0,0218***
0.7 (1 drop)	0.0237****	0,0306****
1.5 (2 drops)	0.0209****	0,0314****
3.6 (5 drops)	0.0211****	0,0274****
7.3 (10 drops)	0.0245****	0,0221****

*, **, ***, **** Nonsignificant differences between Δda values ($p > 0.05$, Student's t test) within lines 1, 2, 3, and 4, respectively.

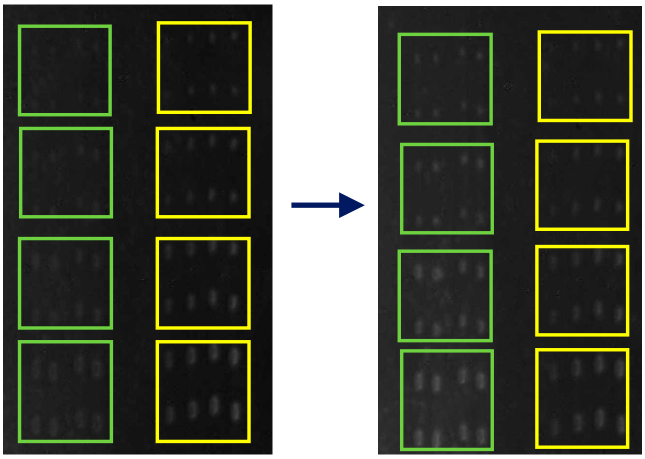

Figure S4. Protein spot images before and during titration with RAD51. The patterns of neutravidin and control immunoglobulin G (IgG) spots are shown in green and yellow frames, respectively.

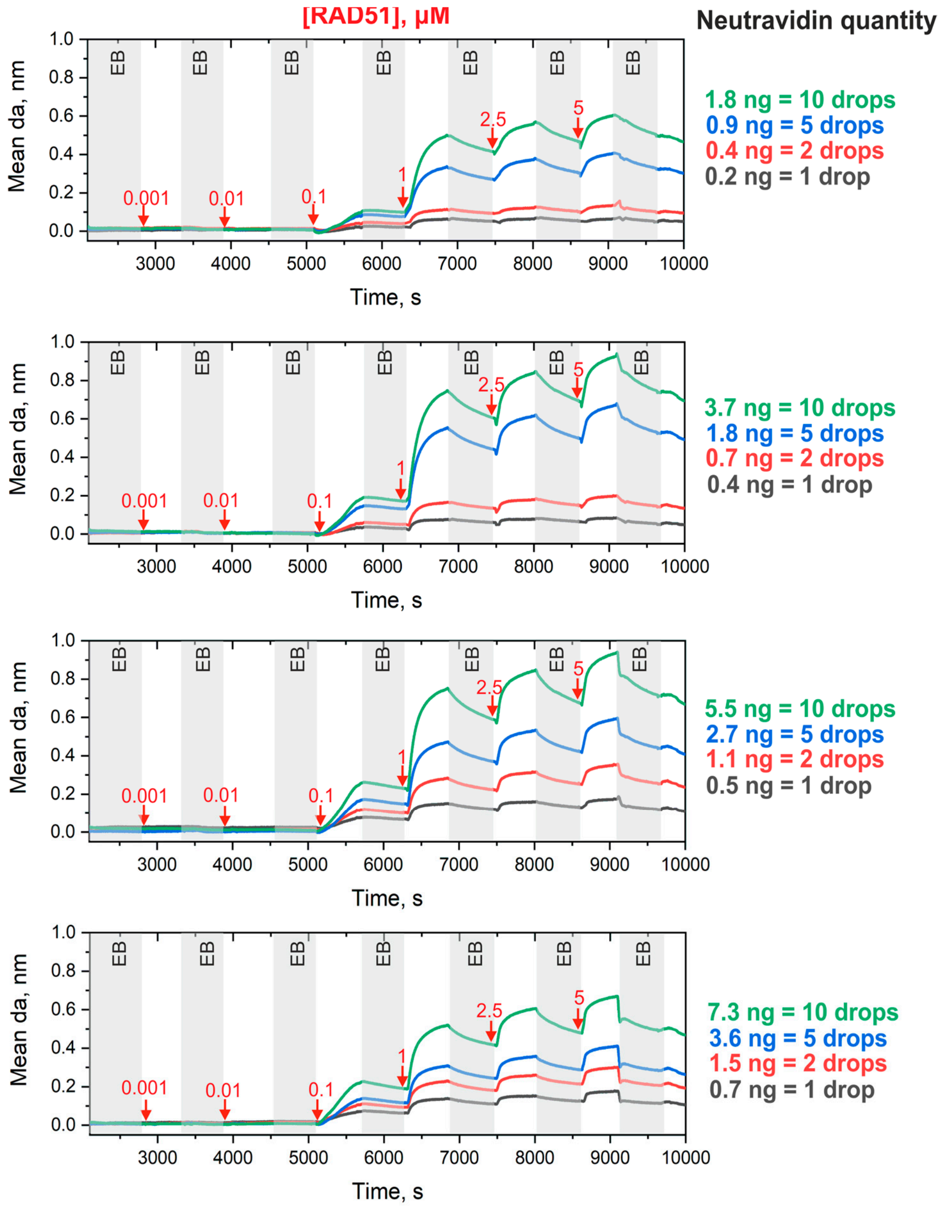

Figure S5. Sensorgrams of RAD51–oligonucleotide association in the selected spots after blank subtraction. The start of the subsequent injections of RAD51 at each concentration is indicated with an arrow. Each sensorgram is an average of two sensorgrams recorded in duplicate. Abbreviations: EB, equilibration buffer; [RAD51], 0.001µM, 0.01 µM, 0.1 µM, 1 µM, 2.5 µM, and 5 µM dilutions of the DNA repair protein.

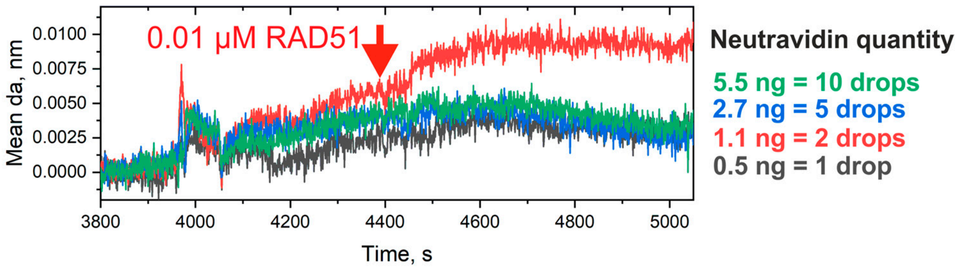

Figure S6. Representative sensorgrams demonstrating RAD51—oligonucleotide association in the selected spots after running solutions containing the RAD51 amount close to the limit of detection. Each sensorgram is an average of two sensorgrams recorded in duplicate after blank subtraction. The arrow indicates the start of the injection of 0.01 µM RAD51.

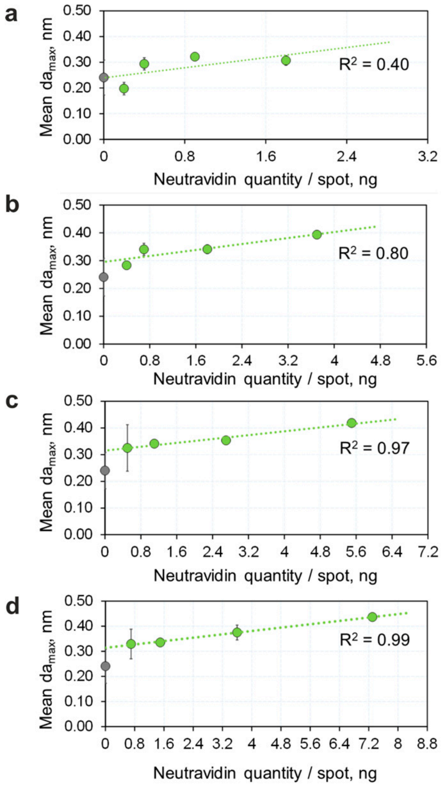

Figure S7. Linearity of the sensor response as a function of the amount of neutravidin applied onto the photonic crystal surface. **(a)** line 1 (0.2, 0.4, 0.9, and 1.8 ng of neutravidin per spot); **(b)** line 2 (0.4, 0.7, 1.8, and 3.7 ng of neutravidin per spot); **(c)** line 3 (0.5, 1.1, 2.7, and 5.5 ng of neutravidin per spot); **(d)** line 4 (0.7, 1.5, 3.6, and 7.3 ng of neutravidin per spot). Abbreviations: R^2, coefficient of determination of the linearity function. The mean values of maximum gains of the adlayer thickness determined over the neutravidin spots are shown with green dots; the mean values of maximum gains of the adlayer thickness determined over the reference areas of the photonic crystal surface not containing protein recognition units are shown with gray dots.
